# Exploring the Relationship between the Caregiver’s Stress Load and Dementia Patient Behavior: A Case Study of Dementia Specialist Outpatient Data from the Southern Medical Center of Taiwan

**DOI:** 10.3390/ijerph17144989

**Published:** 2020-07-10

**Authors:** Cheng-Chang Liu, Chang-Franw Lee, Tung Chang, Jing-Jing Liao

**Affiliations:** 1 Graduate School of Design, National Yunlin University of Science and Technology (YunTech), Yunlin 64002, Taiwan; g9632722@yuntech.org.tw; 2 Doctoral Program, Graduate School of Engineering Science and Technology, YunTech, Yunlin 64002, Taiwan; d10010023@yuntech.org.tw; 3 Department of Business Administration, TransWorld University, Yunlin 64002, Taiwan; j876985@gmail.com

**Keywords:** caregivers, levels of behaviors severity, patient behavior, levels of social care system, long–term care policy recommendations

## Abstract

The aim of this study was to explore the relationship between caregivers’ stress loads and dementia patient behavior, including the correlation of “patient behavior” (severity and frequency), “social care system”, and “stress levels of caregivers”. The research method was based on the analysis of survey data collected at a dementia specialist outpatient clinic of a medical center in southern Taiwan from November 2013 to May 2015. Those surveyed by the center included patients who visited the hospital, and their caregivers completed a questionnaire survey. During the study period, a total of 558 questionnaires for 279 pairs were distributed, and all questionnaires were recovered. According to the survey statistics, the average age of the caregivers interviewed was 53.1 years; women accounted for 61.3% of respondents, and the duration of care exceeded three years. In terms of education, most respondents were college/university graduates. The most common surveyed relationship was that of children acting as the caregiver to a parent, and the average age of the patients was 77.73 years. Most caregivers were found to live with the patients (75.3%). In terms of severity and frequency, the surveyed items with the highest average scores were both the “delusion” item of the “patient behavior” facet, the “mental support”(mean = 1.97; standard deviation, SD = 0.869) item of the “social care system” facet, and the “social life stress” (mean = 2.26, SD = 1.510) item of the “Stress levels of caregivers” facet. The research results show that the “patient behavior” and “Stress levels of caregivers” facets have a significant positive correlation, and the “social care system” and “Stress levels of caregivers” facets have a significant negative correlation. In the future, priority of service planning and implementation of long–term policy should be given to home care, since this is a cultural characteristic of Taiwan. In circumstances where a primary caregiver takes care of family members, the patient’s behavior, length of care, mental support, and social life issues are key items that should be considered in the social welfare control service to alleviate the load of dementia patients on family caregivers.

## 1. Introduction

According to a study by the World Health Organization (WHO), a new case of dementia is diagnosed every three seconds. There were an estimated 47.5 million people worldwide living with dementia in 2015, and this number is believed to be close to 50 million in 2017. The cost of the medical care is over USD 800 billion per year [1].

In 2011, the Ministry of Health and Welfare (MOHW) appointed Taiwan Alzheimer’s Disease Association (TADA) to study dementia epidemiology. In 2016, there were 243,430 dementia patients aged over 65 in Taiwan, which is expected to increase to 670,000 in 2041 (accounting for about 30% of the elder population).

The World Alzheimer Report [2] indicates that the current Dementia Special Outpatient program cannot provide sufficient care for the increasing number of dementia patients. Cooperation between communities and social services is needed to increase the diagnosis rate, lower medical expenses, and provide for the various needs of dementia patients.

Dementia care in Taiwan is similar to that of other Asian countries. Most dementia patients are cared for in the family home or that of a relative. Over 70% of dementia patients are taken care of by a single caregiver or by a spouse [3,4]. An inadequate social care system, slow response to requests for assistance, and low birth rate have led to the burden of care being placed on aging caregivers who are taking care of their elderly relatives.

Modes of care and local customs differ significantly between Eastern and Western societies [5]. In Western societies, elders spend their remaining years in a nursing home, although efforts are being made to have the elderly “age in place” (live at home with assistance from nurses’ aides). In Eastern societies, caregivers would experience negative moral judgements of others if they were to send their elderly relatives to a nursing home [6]. Home is the environment most familiar to patients, providing a sense of comfort and consistency not usually available in an institution (e.g., a nursing home). However, caring for dementia patients at home is an extremely demanding and often exhausting responsibility [7]. Wandering is one of the common behavioral symptoms of Alzheimer’s disease and is the main source of mental stress for caregivers. Patients can easily become lost in time and place, and often try to escape [8,9].

**Figure 1:**
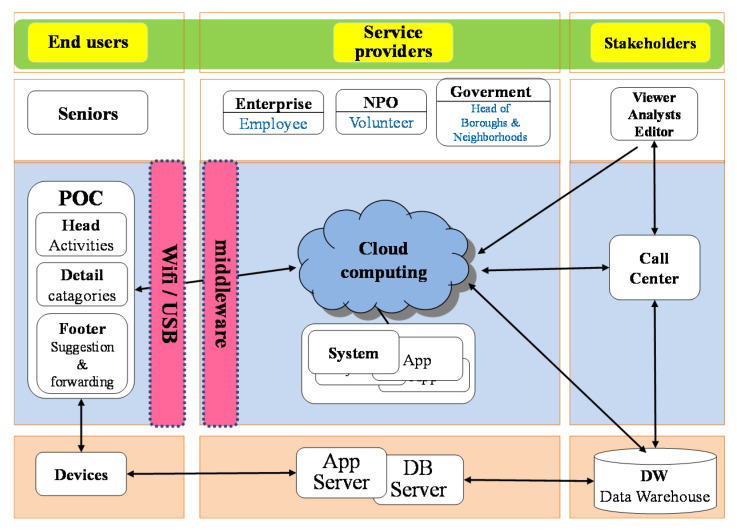
Nursing value chain of caring products and service design for seniors living alone. NPO, Nonprofit Organization; POC, Point of Care; APP, Application; DB, database; DW, Data Warehouse.

Families and caregivers (in Eastern societies) are not professionals. However, they might be able to reduce the incidence of undesired events (a lost or missing patient, for example). Although the Taiwanese government has published its Long–Term Care 2.0 policy, it has not yet been implemented via an industrial value chain (as shown in Figure 1) [10,11]. With such a support system in place, the mental stress of the caregiver could be lowered, and the quality of care increased [12]. Nonprofessional caregivers with sufficient education of the symptoms and behaviors might then be able to evaluate and more effectively respond to the care and safety of their patients. A previous study attempted to create a social caring service framework by combining the design of end user devices, the application of service providers, and the supplier linkages of stakeholders.

Developed countries, such as the United States, Japan, and those in Europe, have studied the issues of ageing in society from the perspective of social welfare. The Taiwanese government has now developed local care policies, such as “Long–Term Care 2.0”. The emotional and mental stress of caregivers can be reduced when they are able to access services and technologies supported by formal, governmental policies. According to the Taiwan Association of Family Caregivers, in 2014, there were a total of 900,000 family caregivers in Taiwan. It was found that caregivers felt stress either directly or indirectly due to the care process. Moreover, stress was related to the caregiver’s background, patient’s disability, and the source of stress (such as knowledge of the disease, level or work stress, financial issues, conflicts in different roles, self–evaluation, and social network support) [9].

The amount of stress inherent in the care process can lead to mental, physical, and social weakness in the providers who care for Alzheimer’s patients. With an effective social care system in place, some of this stress can be reduced. Over the long term, a chronic level of stress cannot be sustained. The inspiration for this study was originally empirical (i.e., life experience). This study explores the connection between the care environment and the stress level of dementia patients and their major caregivers, to better understand the relationship between the stress level of caregivers and dementia behaviors. The goal of this study is to clarify the issues faced by caregivers and provide a reference for the development of long–term service law and implementation of the 10–year Long–Term Policy 2.0. This study could provide a basis for practical treatments by experienced workers who understand the cultural background of home caregivers in long–term dementia families.

## 2. Literature Review

### 2.1. Interactive Theory of Stress and Response Behavior

Stress is a perceptual phenomenon that originates from a psychological process when an individual interacts with the environment. Stress occurs via the evaluation of personal thinking when it causes a personal burden or needs that exceed personal resources and endangers well–being [13]. The stressor is an entity that, when faced with an imbalance of needs from the internal or external environment, affects physical, psychological, and mental well–being, which usually prompts an individual to take action and restore balance.

In the 1960s and 1970s, the theoretical framework of the transactional model of stress and coping proposed by psychologists began to attract more attention. Meaningful stimulation was used to provide patients with behavioral pressure and personal interaction. Through meaningful stimuli, stress interacts with individuals. The core hypothesis is that the process of the individual’s internal interaction and transformation due to the impact of external stress passes through the individual’s primary evaluation of the stressor. Secondary social and cultural resources allow a degree of adjustment, resulting in corresponding behavior. The result of the response is that the effect is manifested in the three aspects of the individual’s emotional comfort, functional status, and health outcomes [1,14]. Coping behavior is a factor that regulates stress response, which can usually be divided into the problem–oriented response and the emotion–oriented response [13].

There are two aspects of evaluation when an individual faces the environment: one is a measure of the ability to show the behavior required by the environment, that is, efficiency expectations; the other is judging whether corresponding behaviors can yield good results, that is, outcome expectations [15]. The former includes the assessment of an individual’s ability and strength and is equivalent to self–efficacy. The higher the self–efficacy, the better the health outcomes. Furthermore, expectations of high self–efficacy will reduce individuals’ negative emotions and lead to better actions, while low self–efficacy can easily lead to escape, defense, and anxiety [16,17].

### 2.2. Stressors, Responses, and Evaluations Affecting Primary Caregivers for Dementia Patients

#### 2.2.1. Psychotic Symptoms of Dementia

Patients with dementia have multiple cognitive impairments according to the diagnostic criteria, including memory impairment, aphasia, apraxia, agnosia, and executive dysfunction, resulting in significant impairment of social and/or professional functions. People with dementia who have behavioral disorders are more troubled by care. Common behavioral disorders include repetitive phenomena, sleep disorders, aggressive behaviors, roaming, getting lost, inappropriate behaviors, bulimia behaviors, and pathological collection behaviors [18,19,20]. These behavioral and psychological symptoms of dementia (BPSD) are not only clinically common (12–70%) but are also the biggest source of stress for the primary caregiver when caring for a patient. Not only does the quality of life of patients and families decline, but families may also send the patients to care facilities earlier, thereby increasing the cost of care [21].

#### 2.2.2. Stress on the Primary Caregiver from Dementia

An article discussing pressure on caregivers states that no disease can place a physical and mental burden on the primary caregiver as heavy as that of dementia [22,23,24]. The stressors of the dementia caregiver include physical pressure, psychological pressure, social pressure, and economic pressure.

Physiological stress includes lack of sleep, poor health, physical changes, fatigue, loss of appetite, loneliness, anger, monotony, impatience, powerlessness, worry about illness, restraint, and depression. Social stress includes reduced social opportunities, lack of recreational activities, inability to meet personal and interpersonal needs, inability to pursue hobbies, and changes in family life. Economic stress includes the need to take leave or resign (i.e., affecting the caregiver’s work), medical hospitalization costs, care and maintenance costs, and home equipment costs [25,26,27]. Positive factors that affect the caregiver’s stress are the current relationship between the main caregiver and the patient, the pre–illness relationship, gender, occupational status, mentality of the caregiver, patient behavior disorder, degree of behavior disorder distress, and social support needs; negatively related variables include daily life function and social support adequacy [22,28,29]. In addition, according to research from related papers in Taiwan, the main characteristics of the caregivers for dementia are the following: (1) spouse is more likely to become a caregiver, (2) 40% of caregivers are over 60 years of age, (3) the health status of most caregivers is relatively poor, (4) and the educational level of caregivers is steadily improving [30,31,32].

#### 2.2.3. Evaluation Factors and Health Outcomes of Caregiver Response Behaviors

The main factors affecting the evaluation of caregiver relatives include the health status of the caregiver, whether they live with the patient, and the patient’s ability to care for daily life [33,34,35]. When expectations are too high, guilt and self–blame can occur, because some caregivers have a negative attitude towards cases of dementia. Some caregivers have a positive attitude towards the patient, but other caregivers are too complex to express their feelings. When caregivers evaluate whether the response behavior is effective, they usually use problem solving, emotional regulation, protection of self–esteem, and social interaction to evaluate the effectiveness of their care. Other studies have noted that the frequency and severity of problem behaviors in elderly dementia patients can affect the caregivers’ evaluation of stress and comfort [36,37]. When the caregivers lack a positive perspective, it is most likely to cause a collapse. Studies have also found that wives and daughters are most likely to suffer from breakdown, and spouses and daughters–in–law are also considered to be at risk [22]. Caregivers can experience restricted social lives, poor physical health, lower life satisfaction, reduced religious participation, reduced vacations, and lack of informational support. As a result, major caregivers are prone to depression [38,39].

**Figure 2:**
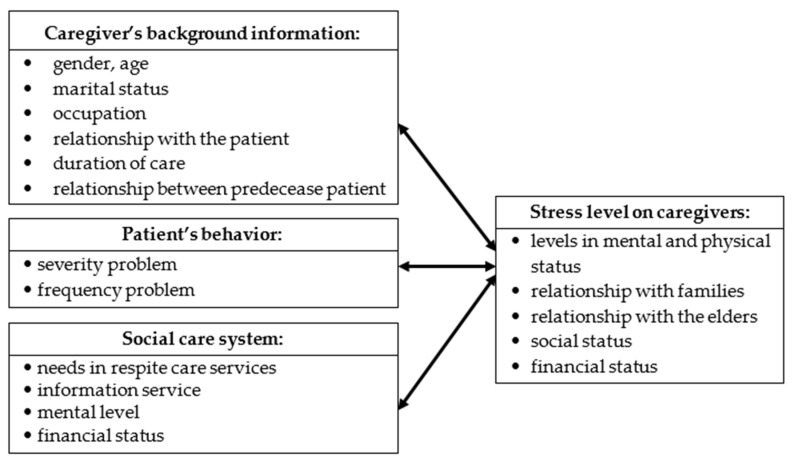
Research structure of the relationship between the stress level of caregivers and dementia patients’ behaviors.

Based on the aforementioned themes and references, this study proposes a research framework that includes an assessment of the basic attributes of the main caregivers of dementia patients, the source of stress (behavior of a dementia patient), and the correlation between the social care system and the stress level of the caregivers (as shown in Figure 2).

## 3. Materials and Methods

### 3.1. Research Content

This research aims to investigate dementia behaviors (severity and frequency), level of the social care system, and the stress levels of caregivers (providers). In addition to collecting information about caregivers through questionnaires, we also used the medical records of neuropsychological examinations of the patients in hospital dementia centers. Moreover, we also collected demographic information, including personal data, occupational history, and family background. Three variables are considered in this study. The measurement methods for the patient and the caregiver are explained as follows:

1. Social care system:

We referred to the family care pressure expansion module [9]. The research examined interviewees’ feelings and internal interactions when they took care of dementia patients from four perspectives. In addition, information about family caregivers and long–term care resources was also explored.

Information relating to the characteristics of the caregiver included their age, gender, area of residence, work status, marital status, and financial status, in addition to the relationship between the caregiver and the patient (spousal caregiver or not), whether living with the patient, having other family members to assist in care, weekly care hours, and long–term resources.

In addition, this study referred to past literature to determine whether caregivers had knowledge of the long–term care services related to local dementia. To define the information relating to long–term care services, this study evaluated information about long–term care resources for caregivers based on caregiver replies to the question “What do you know about the government’s recent provision of resources for long–term care?” Caregiver’s responses were evaluated in the context of six services: (1) community care and care base, (2) home care, (3) home service, (4) daily care, (5) respite service, and (6) family care, etc., items.

2. Stress levels of caregivers:

We based the measurement of caregiver’s stress on the work published by Lee and others [40]. Interviewees’ stress levels were investigated according to timeline, reactions to families, financial situations, and emotions.

Care load is a combination of multi–faceted factors, including physical, psychological, and social aspects. Caregivers must face numerous pressures caused by the long–term care process. Therefore, caregivers are most likely to feel stressed, particularly due to the physical, mental, and social load. The scale most commonly used to assess the load of dementia caregivers is the Zarit Burden Interview (ZBI).

The ZBI scale is designed to measure the subjective feelings of load of home caregivers of dementia patients. The preliminary version of the scale had 29 items, which were subsequently reduced to 22 items. Questions relate to the caregiver’s subjective level of stress and are categorized according to the caregiver’s health status, psychological well–being, finances, social life, and relationships with patients; the survey is mainly carried out by self–assessment.

3. Dementia Patients’ Behaviors:

These were identified using 21 modern dementia outcome measures investigated by Lum and others [41]. Similar to the approach used in the current paper, the previous research used measures of behavior based on frequency (F) and severity (S). Patients’ relationships, needs based on fulfillment, and feedback regarding the patient’s emotional care were also assessed.

Data relating to dementia patients’ behaviors included the patient’s demographic and disease characteristics. The former comprised age, gender, and years of diagnosis. The disease characteristics included three parts: cognitive function, mental behavior symptoms, and daily life features.

Tools for measurement of cognitive functions, namely, the Mini Mental State Examination (MMSE) or Clinical Dementia Rating Scale (CDR), were based on the patient’s examination score that was assessed closest to the time of the caregiver’s interview date. Using the CDR score, the definitions of the severity of cognitive function are 1.0, mild; 2.0, moderate; and 3.0, severe. The National Health Insurance’s standard for the payment of dementia drugs is used to distinguish the severity of dementia based on the MMSE score, which is also affected by the MMSE score. For those with education less than six years, the MMSE group score for mild dementia was adjusted from 15 points or greater to 12 points or greater; moderate dementia was adjusted from 10–14 points to 9–11 points; and severe dementia was adjusted from 9 points or less to 8 points or less.

The measurement tool of patient’s psycho–behavioral symptoms is the Neuropsychiatric Inventory (NPI–10), which measures 10 types of neuropsychiatric symptoms of patients. The test object was the caregiver. The severity and frequency of the patients’ symptoms were evaluated, and the two were multiplied to derive the sub–item score. The maximum total score of the 10–question version of the NPI used in this study was 120 points.

The body function measurement tool uses the Simplified Barthel ADL Index to assess the functions of daily life. It includes 10 functional assessments, including seven self–care abilities and three action abilities. The scoring method has a minimum of 0 points and a maximum of 100 points [21]. The higher the score, the better the patient performs daily activities independently.

The following three criteria were used to analyze the interviewees’ changes in personality: patients’ behaviors, level of social care system, and the stress levels of caregivers.

### 3.2. Research Instruments

#### 3.2.1. Questionnaire

This study questionnaire consisted of four major parts:

1. Demographic information of the interviewees (caregivers and dementia patients);
2. The social care system, which was divided into needs for respite care service, information, mental, and physical;
3. The home caregivers’ stress level, which was divided into five stress dimensions: mental and physical, family relationships, the elders’ relationship, society, and financial status;
4. The patients’ behaviors, which were evaluated in two parts: severity and frequency.

In addition to the collection of demographic information, patients’ behaviors were evaluated using the Neuropsychiatric Inventory (NPI). Frequency was evaluated using a four–point scale. Severity used a three–point scale; other options were valued by a five–point scale. A score of 1 means none, 2–4 means sometimes, and 5 means always.

#### 3.2.2. Validity of Questionnaires

This research instrument was evaluated for content validity (i.e., expert validity). We invited associated experts in the field to evaluate the importance, adequacy, and clarity of the questionnaires and score them using a 5–point scale. Questions scores were sorted by points: very suitable (5 points), suitable (4 points), OK (3 points), not suitable (2 points), and deleted (1 point). The average score from the three expert reviewers was 4.7 points. The questions SD that were greater than 0.5 were modified in phases, and we also removed inappropriate questions.

The questionnaires were simulated before their public release to better understand the interviewees’ beliefs and ideas. Sentences were also revised based on the testers’ advice to avoid confusing statements. The simulation results showed that the simulators understood the questions and special terms, and the questionnaire was judged to be appropriate for distribution.

#### 3.2.3. Research Reliability

We used Cronbach’s α measurement for each questionnaire, achieving an average score of over 0.7 points. To obtain consistency in the internal questionnaire, was interviewed a sample of home caregivers before the questionnaire was released. Based on the interviewee’s feedback, the researchers modified and established the facing each other validity of the questionnaire.

### 3.3. Research Subjects and Study Process

Using both a survey method and a cross sectional study, we sampled the dementia patients of the Dementia Special Outpatient Department in a southern Taiwan medical center. Both the patients and their caregivers were considered research subjects. We cooperated with the department to interview the participants, as it was easier to locate caregivers and dementia patients at the medical center compared to other locations. We obtained the ethical permission of the Institutional Review Board, National Cheng Kung University Hospital (NCKUH IRB) (IRB No: B–ER–102–173). The conditions of selection are as follows:

1. Patients with dementia: Patient must be diagnosed with dementia by a specialist in neurology or psychiatry. Disease subtypes include Alzheimer’s disease (AD), vascular dementia, or mixed dementia. Patients who live in institutions were excluded;
2. Family caregivers: Those who are over 18 years old are related to the patient, have provided care for a minimum of one year, can be interviewed in Mandarin or Taiwanese, voluntarily participated in the study, and signed the research consent.

The selection process involved inviting those who met the inclusion criteria to join the study in a consultation room. After signing the consent form, a telephone interview time was agreed with the caregiver. After obtaining the consent of the nursing staff and the patient, the researchers and the institution signed a cooperation field agreement, and an appropriate time for the primary caregiver to visit the patient was arranged.

We had a field agreement with the medical center and interviewed the primary caregivers of the dementia patients. When the caregivers took the dementia patients to the medical center, we assessed their ability to participate and asked if they would be willing to be subjects in the study. If both the caregivers and dementia patients agreed, they then filled out the questionnaire, and the results were collected for subsequent analysis.

To improve the return and correction rates, we assisted participants in answering each question, one on one. Ten participants answered the questions without a researcher’s assistance. The study period was from November 2013 to May 2015. A total of 558 questionnaires were distributed [42].

### 3.4. Data Analysis

Participant attributes were analyzed based on descriptive statistics using SPSS for Windows version 25. An independent t–test, one–way ANOVA test, and chi–square test were used to identify the demographics. Regression analysis was also used to ascertain the relationship between the dementia patients’ behaviors, level of social care system, and the stress levels of the caregivers based on personal data (gender, age, education).

## 4. Results

Based on the completed questionnaires, the majority of caregivers were female (62.7%). Most dementia patients were cared for by their children (47.0%). The average age of the caregivers was 53.1 years, and up to 34.8% of the interviewees were educated in general and vocational high school. The caregivers’ age ranged from 50 to 59 years (32.62%), and 29.0% of the patients had young onset dementia syndrome (Table 1).

Dementia patients were primarily female (60.6%). The average age of the patients was 77.73 (ranging in age from 49 to 101). The greatest proportion of patients were aged between 80 and 84 (25.4%). The relationships between patients and caregivers were parents (47.0%); couples (39.1%); and couples’ parents (12.2%). The duration of the patients’ education was from 1 to 6 years (45.52%). Patients diagnosed with AD represented 82.4%. The severity level was evaluated as mild/moderate (43.4%) (Table 2). The duration of care, on average, was three years (52.16%). Most often, caregivers and patients lived together (75.3%). The patients or patients’ families absorbed the medical costs, (31.9%) (Table 3).

The researchers evaluated the patient’s behaviors, social care system, and stress levels by scores. The researchers also used demographic information to review the results. Patients’ behaviors were scaled by the Neuropsychiatric Inventory (NPI) on a 5–point scale: 1, rarely; 2–4, sometimes, often, and very often; 5, always.

The average score for severity was 3.39. The highest average score was for “A” delusion, of 1.82. The lowest value 0.46 was for “F” elation. The “E” anxiety value was 1.23, the “I” irritability value was 1.21, the “D” depression and dysphoria value was 1.20, the “J” weird behaviors value was 1.20, the “B” hallucinations value was 1.17, the “C” agitation and aggression value was 1.11, the “G” apathy and indifference value was 0.86, and the “H” disinhibition value was 0.71.

The average frequency score overall was 3.45. The highest score was A “delusion at 1.82. The lowest value, of 0.56, was for “F” elation. The remainder of the values were “E” anxiety, 1.63; “J” weird behaviors, 1.61; “I” irritability, 1.57; “B” hallucinations, 1.56; “D” depression and dysphoria, 1.53; “C” agitation and aggression, 1.37; “G” apathy and indifference, 1.23; and “H” disinhibition, 0.95.

The overall average score in terms of social service support was 1.68. The other scores included respite care, information, mental perspective, and financial support as follows: 1.40, 1.50, 1.97, and 1.91, respectively. Among these scores, mental services were ranked highest followed by financial support and information. Respite care ranked the lowest in terms of need.

The average value for the stress levels of caregivers was 1.28. Mental and physical stress was rated at 1.5, stress of family relationships was 1.17, stress of elders’ relationships was 1.42, stress of social life was 2.26, and the stress of financial status was 0.16.

To summarize, the highest value was on social life stress, followed by mental and physical stress, elders’ relationship, family relationship, and financial status (Table 4).

We also reviewed the degree of severity of the patients’ behaviors, the frequency of patients’ behaviors, and the stress levels of the caregivers. The researchers found a significant relationship between the patients’ behavior and the stress levels of the caregivers (*r* = 0.319 and 0.317, *p* < 0.01). No significant effect was found between the needs in the social care system and the stress levels on the caregivers (*r* = −0.118, *p* < 0.01) (Table 5).

The variables severity of the patients’ behaviors, frequency of the patients’ behaviors, the caregivers’ basic information, and the needs of the social care service system were analyzed using a regression model. The researchers analyzed the influence of the variables on the level of care stress.

The regression results found predictable variables in this study. “People who assist care patients” (*p* < 0.001) and “People who advise the methods on caring for the patients” (*p* < 0.05) showed a significant relationship. In this case, we found that the caregivers felt a lower level of stress.

“Relationship with patients”, “People who could assist with errands or shopping”, and “People who could accompany” (*p* < 0.01) had an insignificant correlation. The level of care stress was higher in the following cases: Caregivers were not close relatives to the patients, no one could assist with errands or shopping, and no one could accompany. The explanatory power was 28.10% of the overall regression mode (Table 6).

## 5. Discussion

In the overall analysis of the basic information, more than half of the caregivers were female, married, had a high school education, and full–time jobs. More than 47% of the caregivers were daughters of dementia patients. Their average age was 53.1 years with a predominant age range of 50–59, 47% of patients were caregivers’ parents, and 60.6% of the patients were female. The predominant age range for dementia patients was 80–84 (more than 25.4%). The study showed that the caregivers and dementia patients were primarily female. The study also indicated that female caregivers had higher stress levels if they did not have assistance with errands, shopping, or accompanying patients.

The study was similar to a national caregivers study conducted by the Taiwan Alzheimer’s Disease Association (TADA). Even when patients were sent to a daily care facility, daughters remained the primary care providers [43]. The research results showed most married females in Taiwan were willing to leave their jobs to take care of their parents. Research also showed a high rate of females will help their siblings in caring for dementia patients.

More than half of the caregivers had been taking care of these patients for over three years (52.16%). The medical expenses were met by the patients themselves or their families (including spouses, 31.9%). Up to 24.0% of the expenses were paid for by the dementia patients’ sons, daughters, and their families.

The caregivers showed a lower stress level in these situations: “People who assist in caring for the patients” (*β* = 0.279, *p* = 0.001) and “People who advise the methods for caring for patients” (*β* = 0.220, *p* = 0.012). When caregivers provided long–term care to dementia patients, their roles in work, family, and society were negatively impacted. Caregivers’ stress levels could be lowered if outside agencies were involved in supporting the caregivers, thereby allowing more time for rest.

This study looked at three major variables: patients’ behaviors, social care system, and the stress levels of the caregivers. We also considered the caregivers’ feedback as a factor in the aforementioned three measures.

The caregivers considered patient behavior to have the highest frequency. In social service support, the score was higher in terms of mental health. For caregiver stress, the score was higher in social stress. With regard to social service support, the interviewees felt that other areas of work were negatively impacted, and more time was occupied with taking care of the patients. With regard to the patients’ behaviors and their severity and frequency, the family caregivers always mentioned the patients’ delusions, anxiety, and abnormal behaviors. These three aspects of the patients’ behavior were the most distressing to the caregivers, increasing their levels of stress.

The difficulty in caring for dementia patients and the stress levels of the caregivers depended on the patient’s condition and course of the disease. Caring for these patients on a daily basis is a constant challenge, particularly when other factors, such as financial pressure, impact on the caregiver. Caring for dementia patients is a constant source of stress for those responsible for their well–being.

When caregivers experience symptoms such as depression and anxiousness, crying, and periods of insomnia, it is clearly important that they seek professional help. Many dementia associations and agencies exist in Taiwan. Both the government and non–governmental organizations (NGOs) have made resources available to both caregivers and patients. Those trained in dementia care (professionals) and experienced caregivers could act as a resource for the caregivers and their families.

This study was based on a sample of primary caregivers and dementia patients registered with a medical center in southern Taiwan. The primary caregivers could have different needs in various social/cultural and medical disease settings. Consequently, this study does not reflect all of the conditions of caregivers’ needs and levels of stress.

In future studies, researchers could investigate caregivers’ reactions to various levels of stress. Moreover, caregivers could find the balance required in families, understand the severity of the diseases, and take steps to manage sources of pressure. Caregivers could learn to prioritize the patients’ behaviors, family needs, and mental health needs [44,45,46,47].

In the future, designers and practitioners could focus more attention on the interactions, family relationships, and social needs of both caregivers and dementia patients. Caregivers could help to improve their patients’ physical health and improve their behaviors. Designers and practitioners could assist family caregivers in evaluating the social service systems and levels of stress. If these two factors could be modified, the stress level of the home caregivers could be reduced.

A final consideration for the future is policy formulation, which should recognize that the needs of caregivers could differ based on the level of stress and the family situation. Policies should therefore adhere closely to the needs of the individual families.

## 6. Conclusions

With regard to aging caregivers in Taiwan, this study identified challenges relating to their finances and physical strength. More community rehabilitation facilities are needed including, but not limited to, daycare, elderly employee counseling, and home recovery. Furthermore, the government should provide more social care services, such as home cleaning, bathing, meal delivery, respite care, and community assistance, in addition to job guarantees and a cost of living allowance. Medical agencies could also have an active role by providing services that lower caregiver stress, education for caregivers’ in the care of dementia patients, and mental health education. Increasing the availability of these resources should be a goal.

This study aligns with the government’s Long–Term Care 2.0 policy to build service integration infrastructure, transfer caregiver experiences and needs in the long–term care process to regional health care institutions, and analyze big data for the design of service products. For caregivers, a point of care (POC) model could be used to record the care process on a smart device and send it to the long–term care system at regular intervals. Through the service integration infrastructure, “Long–Term Care 2.0” will be upgraded to the industrial chain of long–term services.

This study found that advice from clinical practices was critical. It was also important to evaluate the caregivers’ age, education level, physical situation, monthly wage, and care time. Agencies can provide emotional support to caregivers by reducing their feelings of helplessness and stress.

Care agencies should also evaluate the stability of dementia patients and the level of family satisfaction in their deliverance of care. Agencies should schedule multiple programs aimed to create an atmosphere of happiness. Dementia patients could thus experience a more home–like atmosphere. Agencies could also provide guidance on prevention and treatment according to the severity of the patient’s illness.

Taiwan launched a 10–year program in its “Long–Term Care 2.0” policy in 2017. The initial service consisted of respite care for home caregivers only. This was then expanded to eight major services, including mental health support, community support, and technical support. However, due to funding constraints, these eight service items remain inadequate. Among these services, “local creation” and “the elderly are taken care of until death” are only provided by neighborhood agencies. However, most people in Taiwan were not aware of these services, and the long–term care delivery system (the long–term care system and long–term care ABC) was not connected horizontally.

This study found that the interviewed caregivers lacked information about patient care methods, i.e., “people who recommend nursing methods.” It was found that most interviewees needed support services, but they did not have the opportunity to obtain the necessary information. Future study should aim to show that the government and civil society can work together to improve the curriculum for family support skills and home care skills. In addition, “smart homes” can assist in the entire care process, particularly in the case where caregivers may not be able to accompany dementia patients in their homes. The smart home environment is also a long–term service model with significant potential [46]. The related care policy for home caregivers should be more flexible, allowing home caregivers to deploy related resources. In addition, primary caregivers and children of dementia patients would benefit from more supportive policies, including workplace support.

## Figures and Tables

**Table 1:** Basic information of patients and caregivers (*n* = 279).

Variables	*n*	%	Variables	*n*	%
Gender			Caregivers’ Age		
Male	104	37.3	20–29	2	0.72
Female	175	62.7	30–39	16	5.73
Education Level			40–49	41	14.70
Uneducated	12	4.3	50–59	91	36.62
Elementary School	46	16.5	60–69	69	24.73
Junior High School	29	10.4	70–79	30	10.75
High School and Vocational High School	97	34.8	80–89	30	10.75
College	95	34.1	Dementia Stage		
Marital Status			Mild (early stage)	81	29.0
Married	226	81.0	Moderate (middle stage)	73	26.2
Single	45	16.1	Severe (late stage)	46	16.5
Divorced	8	2.9	Unknown	79	28.3
Relationship with Patients					
Spouses	109	39.1			
Parents	131	47.0			
Spouses’ Parents	34	12.2			

**Table 2:** Patients’ basic information (*n* = 279).

Variables	*n*	%	Variables	*n*	%
Gender			Diagnosis Disease Name		
Male	110	39.4	Others	1	0.4
Female	169	60.6	AD Alzheimer’s Disease	230	82.4
Patients’ Age			Dementia	26	9.3
–59	5	1.8	Dementia with Lewy Bodies	10	3.6
60–64	17	6.1	Frontotemporal Dementia	2	0.7
65–69	25	9.0	Parkinson’s Disease with Dementia	3	1.1
70–74	28	10.0	Vascular Dementia	3	2.5
75–79	52	18.6	Early Onset Dementia (EOD)		
80–84	71	25.4	Yes	249	89.2
85–89	55	19.7	No	30	10.8
90–94	22	7.9	Dementia Severity		
95–	4	1.4	Mild	121	43.4
Years of education			Moderate	102	36.6
Uneducated	63	22.58	Severe	56	20.1
1–6 years	127	45.52			
7–9 years	39	13.98			
10–12 years	34	12.19			
13 years or more	16	5.73			

**Table 3:** The relationship between interviewees and patients (*n* = 279).

Variables	*n*	%
**Year(s) that caregivers take care of the patients**		
Less than 1 year	48	17.27
1 year–less than 2 years	44	15.83
2 year–less than 3 years	41	14.75
More than 3 years	145	52.16
**Caregivers live with patients**		
Yes	210	75.3
**Who paid the medical expenses**		
Patients themselves and spouses (patient’s family)	89	31.9
Patients sons’ and daughters’ family	67	24
Patients’ sons and daughters	82	29.4
Patients themselves (divorced)	12	4.3
Single child and his/her family	29	10.4

**Table 4:** Scores among dementia behaviors (severity and frequency), level of social care system, and the stress levels of caregivers.

Variables	Questions	Mean	SD
Severity of Patients’ behaviors	10	3.39	3.002
“A” delusion	1	1.42	1.070
“B” hallucinations	1	1.17	1.116
“C” agitation and aggression	1	1.11	0.997
“D” depression and dysphoria	1	1.20	0.955
“E” anxiety	1	1.23	0.983
“F” elation	1	0.46	0.764
“G” apathy and indifference	1	0.86	1.046
“H” disinhibition	1	0.71	0.988
“I” irritability	1	1.21	1.034
“J” weird behaviors	1	1.20	1.027
Frequency of Patients’ behaviors	10	3.45	3.057
“A” delusion	1	1.82	1.424
“B” hallucinations	1	1.56	1.524
“C” agitation and aggression	1	1.37	1.269
“D” depression and dysphoria	1	1.53	1.230
“E” anxiety	1	1.63	1.320
“F” elation	1	0.56	0.991
“G” apathy and indifference	1	1.23	1.494
“H” disinhibition	1	0.95	1.316
“I” irritability	1	1.57	1.348
“J” weird behaviors	1	1.61	1.382
Needs in Social Care System	15	1.68	0.724
Respite Care Service	5	1.40	0.930
Information Service	3	1.50	0.994
Mental Support	5	1.97	0.869
Financial Support	2	1.91	1.026
Stress Levels of Caregivers	24	1.28	0.650
Metal and Physical Stress	5	1.50	0.807
Relationship with Families	3	1.17	1.136
Relationship with the elders	7	1.42	0.764
Social Life Stress	4	2.26	1.510
Finance Issue	5	0.16	0.278

SD, standard deviation.

**Table 5:** Relationship among patients’ behaviors, social service support, and stress levels (*n* = 279).

Variables	Severity of Patients’ Behaviors	Frequency of Patients’ Behaviors	Needs in Social Care System	Stress Levels of Caregivers
Severity of Patients’ Behaviors	1.000			
Frequency of Patients’ Behaviors	0.999 **	1.000		
Needs in Social Care System	0.093	0.092	1.000	
Stress Levels of Caregivers	0.319 **	0.317 **	−0.118 *	1.000

** *p* < 0.01; * *p* < 0.05, values are correlations.

**Table 6:** Regression analysis of stress levels of caregivers (*n* = 279).

Variables	Standardized Regression Coefficient *β*	Significant Correlation
Severity on patients’ behaviors	0.896	0.462
Frequency on patients’ behaviors	−0.566	0.642
Caregivers basic information		
Gender	0.011	0.870
Education level	0.026	0.721
Marital status	−0.035	0.596
Relationship with patients	−0.186 **	0.010
Year(s) to care patients	−0.002	0.977
Month(s) to care patients	0.064	0.327
Living with patients	0.024	0.729
Paying the medical expenses	0.081	0.190
Dementia types	−0.062	0.306
Needs in social care system		
People who assist with errands or shopping	−0.253 **	0.002
People who help clean	0.005	0.956
People who give financial support	−0.012	0.856
People who assist in caring for patients	0.279 ***	0.001
People who could accompany	−0.234 **	0.005
People who assist in preparing three meals	−0.115	0.206
People who assist in transportation	0.021	0.771
People who give suggestions on finance	−0.033	0.634
People who advise on the methods for caring for patients	0.220 *	0.012
People who remind and advise the caregivers	−0.163	0.067
People who understand the caregivers’ situation	0.052	0.541
People who can be listeners when the caregivers have difficulties	0.050	0.599
People who can be assistants anytime via phone call	−0.146	0.097
People who can cheer up the caregivers	0.000	0.996
People who appreciate and affirm the caregivers	−0.010	0.894
R–Square	28.10%	

*** *p* < 0.001; ** *p* < 0.01; * *p* < 0.05.
